# Head and neck neuroendocrine tumors at a single institution over 15 years

**DOI:** 10.1002/ccr3.2545

**Published:** 2019-11-17

**Authors:** Maria Bacalao, Muhammad S. Beg, Dominick Cavuoti, Hong Zhu, Baran Sumer, Larry Myers, John Truelson, Lucien Nedzi, David Sher, Randall Hughes, Saad A. Khan

**Affiliations:** ^1^ Department of Medicine UT Southwestern Medical Center Dallas Texas; ^2^ Division of Hematology and Oncology/Harold C. Simmons Comprehensive Cancer Center UT Southwestern Medical Center Dallas Texas; ^3^ Department of Pathology UT Southwestern Medical Center Dallas Texas; ^4^ Department of Clinical Sciences UT Southwestern Medical Center Dallas Texas; ^5^ Department of Otolaryngology UT Southwestern Medical Center Dallas Texas; ^6^ Department of Radiation Oncology UT Southwestern Medical Center Dallas Texas

**Keywords:** head and neck cancer, larynx cancer, neuroendocrine cancer

## Abstract

Head and neck cancer is a diverse group of rare diseases such as neuroendocrine tumors which can be thought of as extrapulmonary small‐cell cancer. Surgery, chemotherapy, and radiation can frequently cure this disease, possibly due to early detection.

## Graphical abstract

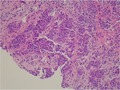

## 1 BACKGROUND

Neuroendocrine head and neck tumors are rare with limited data guiding management. Our institutional series of 11 patients showed age ranges from 21 to 81 years old. Median survival was 21.3 months. Head and neck neuroendocrine tumors can be seen at the extremes of age, and multimodality management can yield good outcomes.

Neuroendocrine tumors of the head and neck are very rare with unclear optimal management strategies. Out of the 1000 cases of extrapulmonary small‐cell carcinoma diagnosed annually in the United States, approximately 60‐120 are in the head and neck region, but the true incidence is unclear.^1^ Risk factors are similar to small‐cell lung carcinoma (SCLC), although recent data suggest that there is less of a correlation with cigarette smoking and extrapulmonary small‐cell carcinoma than there is for SCLC.^1^ In a large case series of extrapulmonary small‐cell tumors, 51% of patients have a history of tobacco use, in comparison with SCLC in which more than 80% of patients have a smoking history.^2^

## 2 PATHOLOGY

Grossly, the tumors often arise in a submucosal location.^3^ Histologically, the cells usually appear small and hyperchromatic with scant cytoplasm, especially in the oat cell type. An intermediate type has been characterized as well, with a similar growth pattern but with more abundant cytoplasm. Areas of necrosis and high mitotic activity are common.^3^

In young adults, nonhematologic tumors of the head and neck region are rare. We review all cases of head and neck neuroendocrine cancer in the cancer registries of UT Southwestern Medical Center and Parkland Hospital. We report the case of a patient with a neuroendocrine tumor of the larynx and their outcome. To our knowledge, this is the youngest reported case of head and neck neuroendocrine tumor in the literature.

## 3 CASE

The patient is a 21‐year‐old woman who presented with a 6‐month history of progressive hoarseness and dysphagia for solids worse than liquids and a 15 lb weight loss. She had a 3 pack‐year smoking history and did not use alcohol. On physical examination, there was palpable, tender left cervical adenopathy in levels III and IV, largest measuring 2 cm.

**Figure 1:**
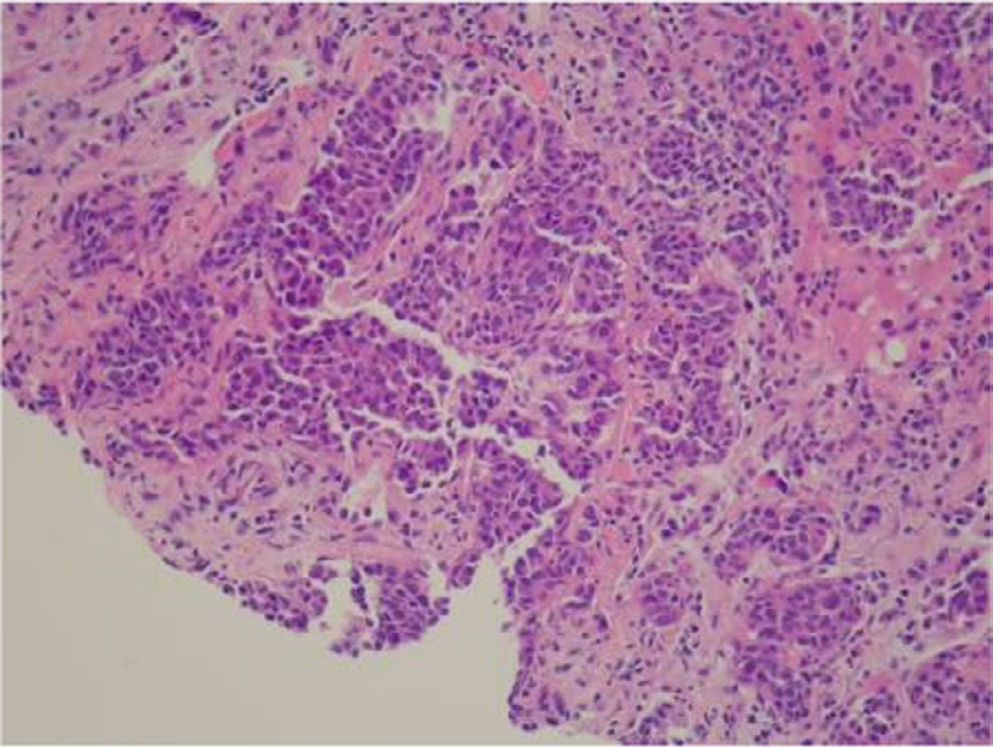
H&E stain of liver metastasis at 20× magnification

**Figure 2:**
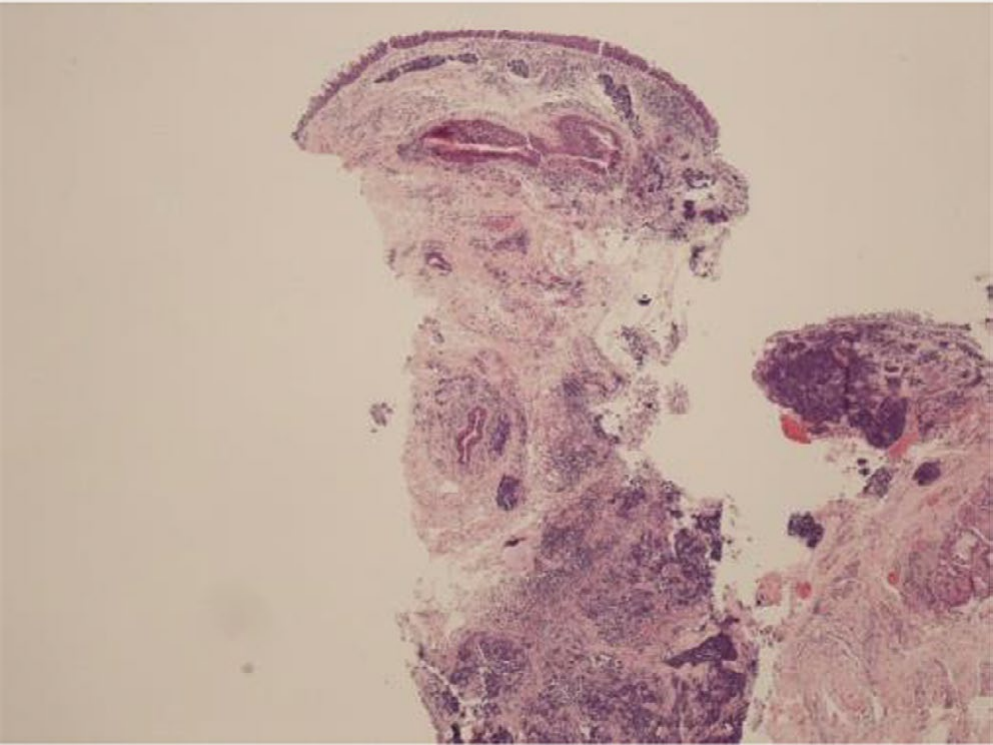
H&E stain of primary laryngeal tumor at low magnification

Computed Tomography (CT) of the neck showed a locally advanced supraglottic mass extending from the vallecula along the AE folds to the true vocal cords. It eroded the thyroid cartilage and extended into the strap muscles. Biopsy of an enlarged left cervical lymph node showed chromogranin and synaptophysin positivity with a high Ki67; CK 5/6 was negative. The tumor was described as poorly differentiated though no formal grade assigned. The initial diagnosis was unclear and a repeat biopsy of the supraglottic mass showed positivity for CKAE1/AE3 and synaptophysin. It was focally positive for CD99 and negative for chromogranin, CK5/6, SALL4, desmin, and TTF‐1. Stains for CD31 and D2‐40 localized to the lymphovascular spaces. There was no evidence of EWSR1 (22q12) rearrangement. Figures 1 and 2 show biopsies from the liver metastasis and larynx.

CT of the chest did not reveal any suspicious nodularity or evidence of metastases. MRI scan showed multiple liver lesions which on biopsy were consistent with metastatic neuroendocrine carcinoma. She was diagnosed as having a T4N2M1 neuroendocrine tumor of the supraglottis. A tracheostomy tube was placed for airway protection.

She was started on etoposide and cisplatin, but developed neutropenic fever with negative cultures shortly afterward, later developing intractable nausea and vomiting requiring hospitalization. After 2 cycles, she was switched to carboplatin and etoposide. In spite of these changes, she still could not tolerate chemotherapy. She subsequently developed neutropenic fever, requiring a lowering of the dose and the addition of pegfilgrastim. Repeat CT of the neck conducted shortly before the second carboplatin/etoposide cycle showed that the supraglottic tumor size was unchanged, but that the associated lymphadenopathy at the level 3 lymph nodes had improved. She did not tolerate chemotherapy and discontinued all therapy, expiring shortly thereafter.

## 4 METHODS

IRB approval was obtained from the institution for this work. We searched the cancer registries of UT Southwestern and Parkland Memorial Hospital for patients with small‐cell carcinoma or neuroendocrine carcinoma in the head and neck from 1997 to 2014. The overall survival was estimated by the Kaplan‐Meier analysis, and we compared the overall survival between the UT Southwestern University Hospital and Parkland Hospital by the log‐rank test. A *P*‐value of <.05 indicates the statistical significance. We used Stata software for analysis.

## 5 RESULTS

A total of 11 cases of head and neck neuroendocrine tumors were identified: 7 from Parkland Memorial Hospital and 4 from UT Southwestern. Of the Parkland patients, 5 were male and 2 were female, consistent with the male predominance noted in the other case studies and meta‐analyses.^2^, ^4^ Although the Parkland system treats many Hispanic patients, only one patient in the series was of Hispanic origin. Most of the patients presented with locoregionally advanced disease, and 3 had metastases present at diagnosis. For treatment, two patients received surgery alone and two received chemotherapy alone. Three patients received combination therapy: one received chemoradiation, another received chemotherapy and surgery, and a third received radiation and surgery. Overall, this represents a higher percentage of patients receiving monotherapy than expected based on these other series, considering the advanced stage of the disease.

An additional 4 cases were seen from the registry at UT Southwestern. These patients were significantly older (68‐81 years, mean 75) and had less advanced disease. Only 2/4 patients received chemotherapy, and all received surgery, either for an excisional biopsy or definitively. In this dataset, histologic grade of the tumor was not available. At the time of analysis, only the eldest patient had deceased, and others were listed in our registry as living.

Our institutional experience shows that these tumors also occur in other sites such as the parotid and nasopharynx. There was one surviving patient in his early twenties, and the key factor in achieving long‐term survival appeared to be early intervention. Table 1 details the patients that were analyzed.

**Table 1:** Patients presenting to Parkland and UT Southwestern with small‐cell/neuroendocrine tumor of the head and neck region

Age at diagnosis	Race	Sex	Primary site	Listed AJCC stage	Modality	1st course Rx summary	Date of diagnosis to last contact	Vital status
Parkland hospital cases								
63	Black	M	Glottis	4A (T4a N0 M0)	Small‐cell carcinoma, NOS	Surgery, Radiation	1/6/2013‐05/15/2013	Deceased (cancer)
68	Black	M	Supraglottis	4C (T2 N2c M1)	Small‐cell neuroendocrine carcinoma	Chemotherapy	2/29/12‐11/28/2013	Deceased (cancer)
56	White	M	Tonsillar fossa	Not available	Combined small‐cell carcinoma	Surgery, radiation, chemotherapy	10/26/2001‐07/11/2003	Deceased (cancer)
47	White	M	Parotid gland	4 (T1 N2 M0)	Neuroendocrine carcinoma, NOS	Surgery	6/16/1997‐02/01/2014	Alive
23	White, Hispanic	M	Nasal cavity	Not available	Neuroendocrine carcinoma, NOS	Radiation and chemotherapy	11/12/1999‐07/07/2003	Alive
21	White	F	Supraglottis	4C (T4a N2 M1)	Neuroendocrine carcinoma, NOS	chemotherapy	5/20/2014‐02/05/2015	Deceased (cancer)
44	White	F	Nasal cavity	4C (T3 N2c M1)	Neuroendocrine carcinoma, NOS	Surgery	4/20/2011‐07/14/2011	Deceased (cancer)
UT Southwestern cases								
81	White	M	Parotid gland	4A (T4a N2 M0)	Small‐cell carcinoma, NOS	Surgery	09/16/2003‐12/18/2003	Deceased (cancer)
68	White	F	Nasopharynx	2 (T1 NX M0)	Adenocarcinoma with neuroendocrine differentiation	Surgery, radiation	02/13/2006‐01/24/2014	Alive
78	White	M	Parotid gland	Not available	Primitive neuroectodermal tumor, NOS	Surgery, radiation, chemotherapy	12/14/2011‐06/18/2014	Alive
71	White	F	Tongue anterior 2/3	1 (T1 N1 M0)	Neuroendocrine carcinoma, NOS	Surgery, radiation, chemotherapy	01/31/2012‐02/11/2014	Alive

In our cohort, the most common site was the parotid gland with 3/11 (27.3%). For all the patients, median overall survival was 21.3 months. Survival did not significantly differ between patients treated at the university compared to Parkland Hospital (log‐rank *P* = .240). In our limited sample, no statistically significant survival variation was noted by race, primary site, or age.

## 6 DISCUSSION

The biology of neuroendocrine tumors of the head and neck is being better understood, and factors which predict for survival are being recognized. Neuroendocrine tumors show higher levels of hypoxia‐inducible factor 1‐α (HIF‐1α), the cellular glucose uptake transporter (GLUT‐1), and the phosphatidylinositol 3‐kinase (PI3K)/protein kinase B (AKT), which may regulate GLUT‐1 and HIF‐1α^6^ than precancerous lesions. The prognostic significance of this remains unclear. Known poor prognostic factors include distant metastases^6^, ^7^ and ectopic hormone production.^6^, ^8^ There does not appear to be a correlation between tumor size or number of mitoses and survival.^6^, ^7^

## 7 TREATMENT RECOMMENDATIONS

The treatment of extrapulmonary neuroendocrine tumors depends on whether the tumor is resectable, locoregionally advanced but unresectable, or metastatic. Treatment options include surgical resection follow by chemotherapy and/or radiation; but if the tumor is unresectable then chemotherapy and radiation is used. Patients with small‐cell tumors are more likely to receive chemoradiation as the primary treatment modality, while those with other neuroendocrine tumor types are more likely to undergo surgical resection.^4^ For small‐cell head and neck tumors, chemotherapy with a small‐cell lung cancer regimen such as cisplatin or carboplatin with etoposide is used regardless of whether the tumor is resected as recommended in the NCCN guidelines. A small case series of 14 patients with head and neck small‐cell cancer showed 1 patient receiving surgery alone as treatment,^2^ while a larger meta‐analysis consisting of patients with laryngeal small‐cell carcinoma showed 14% of such patients receiving surgery alone.^4^ Radiation therapy plays an important role as well, as a case series of 12 patients from the University of Miami showed a 73% 1‐year survival rate in those receiving radiation or chemoradiation vs 67% in those undergoing other treatment modalities.^5^ Overall, the most common treatment modality for small‐cell head and neck tumors remains chemoradiation, received by 34.5% of the patients studied by van der Laan.

## 8 CONCLUSIONS

Head and neck neuroendocrine tumors remain a rare condition, and therefore, it is unlikely that prospective randomized control trials will ever provide clear guidance as to optimal treatment. However, the reported cases provide retrospective data regarding the epidemiology, treatment, and outcomes of small‐cell carcinoma in the head and neck.

These tumors respond to the same chemotherapy and radiation regimens used for small‐cell lung cancer. Unlike small‐cell lung cancer, small‐cell head and neck tumors are sometimes managed surgically, though rarely as the sole treatment modality.

Five out of 11 patients were alive at the time of analysis. This better‐than‐expected survival may reflect a more aggressive treatment approach at our institution or a better prognosis compared to other extrapulmonary small cells, perhaps due to earlier opportunities for detection. Even in cases where chemotherapy was not administered, a young patient was still living more than 15 years from diagnosis. Our series provides additional data about this rare disease and how it can affect very young adults as well. The anatomic distribution varied; the parotid was the most commonly involved site. Nearly all patients listed as alive had surgery, though long‐term survival was noted in one case with combined radiation and chemotherapy. This series should also lead to future evaluation of racial predisposition toward neuroendocrine cancer. Though treating doctors at both institutions are the same, 85% of the patient population at Parkland Hospital self‐identifies as an ethnic minority. Out of our 7 patients at Parkland Hospital, 5 were white; while all of the patients in the University Hospital were white. An analysis of head and neck small cases from the SEER‐Medicare database^9^ found that 86% occurred in white patients.

Limitations of this analysis include those inherent to retrospective studies, such as inability to collect histologic differentiation data in all but the most recent patient. Poorly differentiated neuroendocrine tumors are expected to have worse outcomes, but that information was not available in this analysis.^10^ The small sample size makes it difficult to develop meaningful conclusions.

This series represents one of the larger institutional reports on small‐cell neuroendocrine cancer and suggests that with an aggressive multimodality approach, long‐term survival is a possibility.

## CONFLICT OF INTEREST

None declared.

## AUTHOR CONTRIBUTIONS

All authors were involved in the following aspects of this work: substantial contribution to the study concept and design or acquisition, analysis, or interpretation of the data, and drafted or critically revised the manuscript's intellectual content. All authors approved the final version for publication and agreed to be accountable for all aspects of the work, as well as ensuring all questions related to accuracy and integrity of this work are appropriately answered.
